# New insight into the microbiome, resistome, and mobilome on the dental waste water in the context of heavy metal environment

**DOI:** 10.3389/fmicb.2023.1106157

**Published:** 2023-04-20

**Authors:** Xiaoyang Jiao, Wenyan Guo, Xin Li, Fen Yao, Mi Zeng, Yumeng Yuan, Xiaoling Guo, Meimei Wang, Qing Dong Xie, Leshan Cai, Feiyuan Yu, Pen Yu, Yong Xia

**Affiliations:** ^1^College of Medicine, Shantou University, Shantou, China; ^2^Department of Clinical Laboratory, First Affiliated Hospital of Shantou University Medical College, Shantou, China; ^3^Department of Pharmacology, College of Medicine, Shantou University, Shantou, China

**Keywords:** dental wastewater, resistome, mobilome, antibiotic resistance genes, antimicrobial resistance

## Abstract

**Object:**

Hospital sewage have been associated with incorporation of antibiotic resistance genes (ARGs) and mobile genetic elements (MGEs) into microbes, which is considered as a key indicator for the spread of antimicrobial resistance (AMR). The compositions of dental waste water (DWW) contain heavy metals, the evolution of AMR and its effects on the water environment in the context of heavy metal environment have not been seriously investigated. Thus, our major aims were to elucidate the evolution of AMR in DWW.

**Methods:**

DWW samples were collected from a major dental department. The presence of microbial communities, ARGs, and MGEs in untreated and treated (by filter membrane and ozone) samples were analyzed using metagenomics and bioinformatic methods.

**Results:**

DWW-associated resistomes included 1,208 types of ARGs, belonging to 29 antibiotic types/subtypes. The most abundant types/subtypes were ARGs of multidrug resistance and of antibiotics that were frequently used in the clinical practice. *Pseudomonas putida*, *Pseudomonas aeruginosa*, *Chryseobacterium indologenes*, *Sphingomonas laterariae* were the main bacteria which hosted these ARGs. Mobilomes in DWW consisted of 93 MGE subtypes which belonged to 8 MGE types. Transposases were the most frequently detected MGEs which formed networks of communications. For example, ISCrsp1 and tnpA.5/4/11 were the main transposases located in the central hubs of a network. These significant associations between ARGs and MGEs revealed the strong potential of ARGs transmission towards development of antimicrobial-resistant (AMR) bacteria. On the other hand, treatment of DWW using membranes and ozone was only effective in removing minor species of bacteria and types of ARGs and MGEs.

**Conclusion:**

DWW contained abundant ARGs, and MGEs, which contributed to the occurrence and spread of AMR bacteria. Consequently, DWW would seriously increase environmental health concerns which may be different but have been well-documented from hospital waste waters.

## Introduction

Hospital wastewater is a major “breeding” ground for various pathogens, antibiotic resistance bacteria (ARB) and antibiotic resistance genes (ARGs), and has generated continued environmental health concerns (Rizzo et al., 2013). The major reasons for the concerns are that the wastewater facilitated ARG-exchange events among bacteria and generation of multi-drug resistant (MDR) bacteria (Bondarczuk et al., 2016). However, similar concerns for dental waste water (DWW) have not been specifically investigated.

The DWW has some specific differences from that of hospital sewage. For example, certain oral bacteria (i.e., *Pseudomonas. gingivalis*) were associated with development of oral and gastrointestinal cancers (Ahn et al., 2012; Olsen and Yilmaz, 2019). DWW contains non-infectious toxic wastes that include acrylic resin scraps, metal alloys, porcelain, gypsum and dental amalgam, as well as abundant heavy metals, such as mercury, silver, tin, zinc, and copper which have toxic properties (Clarkson et al., 2003; Jones, 2004; Kao et al., 2004; Vandeven and McGinnis, 2004). Consequently, most investigations on health hazards from DWW have been focused onto amalgam and other metals (Al-Khatib and Darwish, 2004; Muhamedagic et al., 2009), and acrylic resin filling materials (Binner et al., 2022). However, investigations using holistic and more sophisticated technologies have not been reported yet.

Metals and biocides may co-select for antimicrobial resistance (AMR; Gelalcha et al., 2017; Pal et al., 2017). Metal contaminations have been reported to significantly influence the diversity, abundance and mobility potential of a broad spectrum of ARGs in urban soils (Song et al., 2017; Zhao et al., 2019). In addition, co-selections of antibiotic-and metal-resistance have been associated with arsenic (As), cadmium (Cd), cobalt (Co), chromium (Cr), copper (Cu), mercury (Hg), nickel (Ni), lead (Pb), and zinc (Zn) (Pal et al., 2015; Song et al., 2017; Zhao et al., 2019). Another study showed that metal contamination in soil increased the potential for horizontal gene transfer (HGT) of ARGs *via* co-selection of ARGs and MGEs, thereby generating a pool of high-risk mobile ARGs (Martínez et al., 2015). With the presence of heavy metals in DWW as opposed to hospital waste water, DWW may involve novel mechanisms for ARGs evolution, HGT development and transmission of AMR. Unfortunately, there has not been a report on such investigation, especially using resistome and mobilome.

Standard handling and disposal of potentially infectious and toxic DWW has been implemented. Many dental clinics have chair-side primary and secondary filter traps which remove approximately 60% of large particles from discharges (Westman and Tuominen, 2000; Johnson and Pichay, 2001; Adegbembo et al., 2002). In addition, membrane bioreactors (MBR) in combination with biological degradation and membrane separation, have been used to remove infectious and non-infectious agents from effluents (Diehl and LaPara, 2010; Ju et al., 2016). To our knowledge, there has been no reports simultaneously identifying the bacterial communities, resistome and mobilome in DWW. Thus, the overall aim of our study was to investigate the abundance and components of bacterial communities, ARGs and MGEs in treated and untreated DWW from a single dental department. The investigation utilized advanced metagenomic and bioinformatic methods to provide in-depth characterizations of the DWW. Our investigation provides novel information on AMR evolution under high metal pressure and on environmental health concerns.

## Methods

### Dental waste water treatment and sample collection

The DWW samples from each washbasin or dental chair in the department were discharged *via* pipes with filters, to remove large particles, and then into a regulating pool in a tank outside the department. In the tank, the discharged water was homogenized and when the accumulated DWW reached a certain level, it triggered a high voltage discharge which produced ozone and activation of a lift pump which circulate the waste water. After the lift pump stopped working, ozone disinfection continued for another 20 min. In addition, the tank was regularly disinfected once a week by adding chlorine dioxide tablets 5–10 tablets/time (chlorine content 10%) for 30 min.

DWW samples (untreated and treated) of 1 liter each were collected from the specific discharge from the dental department (without mixing with discharge from other sources), weekly from June to July 2021. A total of nine samples were collected in sterile bottles and delivered on ice to the diagnostic microbiology laboratory within 1 h. In the laboratory, each sample was centrifuged at the speed of 10,000 rpm for 5 min at 4°C. The sediments were stored at −80°C until further analysis.

### Metagenome sequencing (DNA extraction and identification)

Microbial DNAs from the sewage sediments were extracted using the E.Z.N.A.® soil DNA kit (Omega Bio-Tek, Norcross, GA, United States). DNA concentrations were measured by using the Qubit® dsDNA Assay KitinQubit® 2.0 Fluorometer (Life Technologies, CA, United States), and about 1 μg of DNA (OD: 1.8–2.0) from each sample was used to construct a library. Sequencing libraries were generated using NEB Next®Ultra™ DNA Library Prep Kit for the Illumina (NEB, United States) analysis, and libraries were analyzed using the Agilent 2,100 Bioanalyzer and quantified using PCR. The thermal cycling conditions consisted of initial denaturation at 98°C for 30 s, 12 cycles of 98°C for 10 s, 65°C for 75 s; and a final extension of 5 min at 65°C. Clustering of the index-coded samples were performed on a cBot Cluster Generation System. After the cluster generation, the library preparations were sequenced on an Illumina platform, and paired-end reads were generated. The bacterial genomic sequences were deposited in the NCBI Sequence Read Archive with an accession number (PRJNA869027) which can be shared with readers.

### Raw sequence pre-processing

The raw data obtained by sequencing using the Illumina sequencing platform has a certain percentage of low-quality data, and in order to ensure accurate and reliable results for subsequent analysis, the raw sequencing data need to be preprocessed, including quality control [Trimmomatic (v 0.39; Sewe et al., 2022) parameter: ILLUMINACLIP: adapters _path:2:30:10 SLIDINGWINDOW:4:20 MINLEN:50], and de-hosting sequences (Bowtie2 parameter: --very-sensitive) to obtain clean data for subsequent analysis. The key parameters are explained below: removal of splice sequences (parameter ILLUMINACLIP: adapters_path:2:30:10); scanning sequences (4 bp sliding window size) and excising subsequent sequences if the average quality score is below 20 (99% correct; parameter SLIDINGWINDOW:4:20); and removing sequences with a final length of less than 50 bp (parameter MINLEN:50).

### Bioinformatics analyses

Short-read sequencing data were used to identify MGEs and ARGs by the Comprehensive Antibiotic Resistance Database protein homolog model version 1.1.2 (CARD; McArthur et al., 2013) and the ResFinder version 2.1. The MGE database is available from https://github.com/KatariinaParnanen/Mobile Genetic Element Database. Once ARGs and MGEs were identified within assembled contigs, the next step involved identifying which contigs contained both ARGs and MGEs. Co-occurring placements within a single contig were considered as evidence for putative genomic colocalization (Paetzold et al., 2019). Reads were assembled individually into contigs by using MEGAHIT (v 1.1.1), with the following parameters: -k-list 39, 49, …, 129, 141 -mincontig-len 1,000. The qualities of assemblies were evaluated by using QUAST (v 5.0.2; Gurevich et al., 2013). The ORFs on ACCs were annotated or retrieved in the CARD database by using Bowtie (2-2.2.9). According to the result of CARD annotation, MGEs which were located on ACCs were identified in the MGE database by using Bowtie (2-2.2.9). Annotations were categorized as MGEs based on string matches to one of the following keywords (Langmead and Salzberg, 2012).

### Network analyses

Network analyses were performed with R using the Vegan and Hmisc packages, and visualizations were conducted on the interactive platform of Gephi 0.9.2. Ggplot2 and pheatmap packages were used to draw a clustering heatmap of ARGs abundance in the samples, and the Hmisc package was used to calculate the correlation matrix for making the network map (Feng et al., 2015). Spearman’s rank correlations were used to construct the co-occurrence networks between ARGs and MGEs, ARGs subtypes and microbial communities that occurred in at least 80% of all samples (Karaolia et al., 2021). A correlation between any two items was considered statistically significant if Spearman’s correlation coefficient (ρ) was ≥0.7 and the value of *p* was <0.001.

## Results

### Diversity of bacterial community, ARG, and MGEs in the DWW

**Figure 1:**
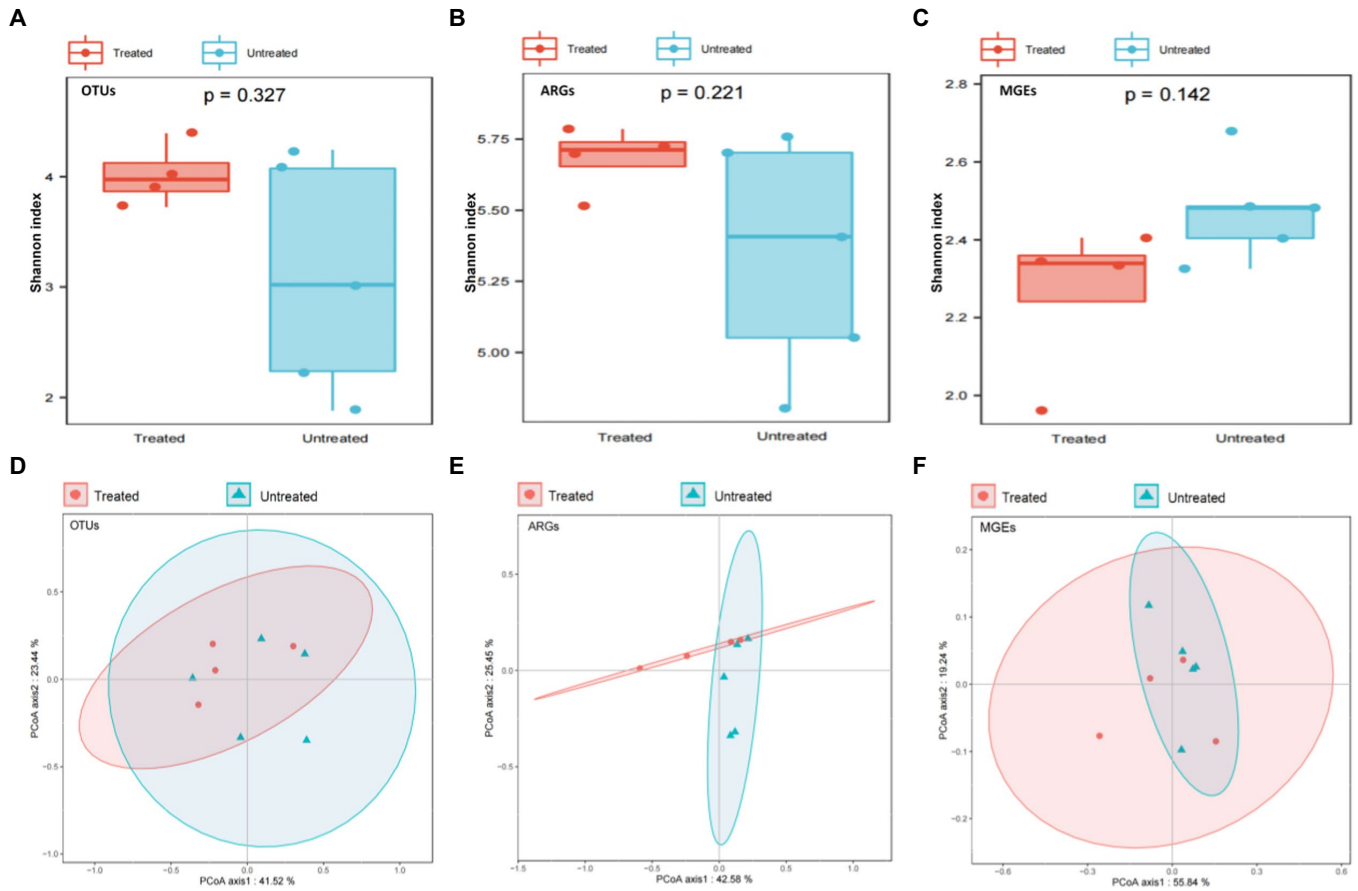
Comparison of diversities among OTUs, ARGs and MGEs in the untreated and treated dental waste waters. **(A)** alpha-diversity of OTUs; **(B)** alpha-diversity of ARGs; **(C)** alpha-diversity of MGEs; **(D)** Beta diversity of OTUs; **(E)** Beta diversity of ARGS; **(F)** Beta diversity of MGEs.

Characteristics of bacterial communities in both the untreated and treated groups of DWW were determined. Alpha diversity including Shannon index/diversity, Simpson index/diversity, richness index and evenness index showed a similar trend between the two groups of DWW. Thus, the Shannon diversity was selected as representative of the alpha diversity (Leiviska and Risteela, 2022) and there was no significant difference between the two groups of DWW samples (*p* > 0.05). For example, the diversity of bacteria and ARGs was insignificantly higher in the treated sewage than in the untreated group, while the diversity of MGEs was insignificantly lower in the treated than that in untreated sewages (Figures 1A–C).

Beta diversity was used to reveal differences in species complexity. The principal coordinate analysis (PCoA) based on Bray-Cutis distance was used to analyze the Beta diversity of OTUs, ARGs and MGEs in both DWW samples, and the PERMANOVA analysis to check whether there was a significant difference in community composition structures between the two groups. The results show that there was no significant difference in bacteria, ARGs and MGEs composition between the two groups. The PCA analyses show that the standard treatment of DWW did not appear to have significant impact on the microbial communities in the waste water (Figures 1D–F).

### Microbiome in the DWW

**Figure 2:**
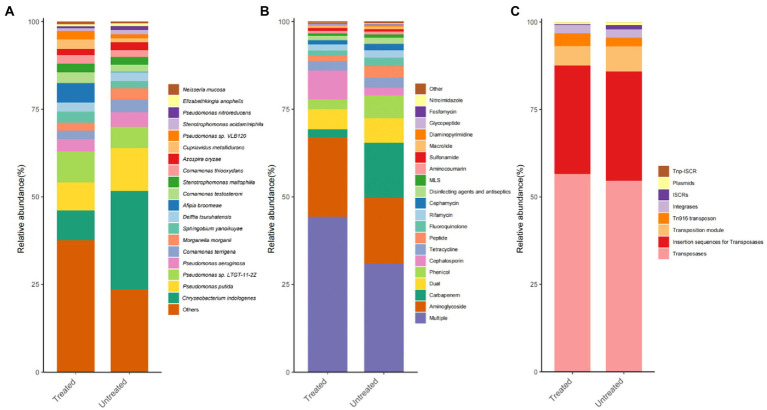
Histograms of the relative abundance distributions of the TOP 20 dominant species, ARGs and MGEs in dental waste waters. The abscissa represents the sample groups, and the ordinate corresponds to the proportion of dominant species. **(A)** Bacteria; **(B)** ARGs; and **(C)** MGEs. The color order from top to bottom of the histogram corresponds to the color order of the legend on the right.

In total, 1, 574 microbial species were identified in the DWW. Among them, there were 1,514 types of bacteria (99.89%), 37 types of fungi (0.093%), 11 types of phages (0.003%), 7 types of Archaea (0.012%), and 5 types of viruses (0.002%). Then, we focus on the bacteria as it is the most abundant component. A total of 4 bacterial phyla with relative abundance of over 1% were identified. The most abundant phyla were Proteobacteria (62.27%), followed by Bacteroidetes (26.66%), Actinobacteria (4.93%), Firmicutes (4.62%), and other phyla (1.52%). In the general level, the most abundant genus was *Pseudomonas* (25.67%), followed by *Chryseobacterium* (19.43%), *Comamonas* (7.98%)*, Stenotrophomonas* (3.33%), *Delftia* (3.07%), *Sphingobium* (2.80%), *Morganella* (2.77%)*, Afipia* (2.66%)*, Prevotella* (2.41%)*, Azospira* (2.03%)*, Cupriavidus* (1.91%)*, Streptococcus* (1.48%), *Aeromonas* (1.42%)*, Elizabethkingia* (1.42%)*, Neisseria* (1.30%), and *Actinomyces* (1.17%). Among bacterial species, the most abundant species was *Chryseobacterium indologenes* (19.32%), followed by *Pseudomonas putida* (10.32%), *Pseudomonas* sp. *LTGT-11-2Z* (7.35%), *Pseudomonas aeruginosa* (3.88%), *Comamonas terrigena* (3.07%), *Morganella morganii* (2.77%), *Sphingobium yanoikuyae* (2.55%), *Delftia tsuruhatensis* (2.54%), *Afipia broomeae* (2.52%), *Comamonas testosterone* (2.50%), *Stenotrophomonas maltophilia* (2.32%), *Comamonas thiooxydans* (2.18%), *Azospira oryzae* (2.03%), *Cupriavidus metallidurans* (1.85%), *Pseudomonas* sp. *VLB120* (1.73%; only presented bacteria with relative abundance over 1%; Figure 2A).

### Variations and relative abundances of ARGs and MGEs types/subtypes in the DWW

The abundance and structure of ARGs were measured. In total, 1,208 types of ARGs were found, belonging to 29 antibiotic types/subtypes. Among them, the most abundant types or subtypes were multi-drug resistant (523), Aminoglycoside (96), Cephalosporin (95), Fluoroquinolone (86), Tetracycline (46), Peptide (37), Cephamycin (36), Phenicol (35), Glycopeptide (32), Carbapenem (20), Diaminopyrimidine (17), Rifamycin (14), Macrolide (12), Penam (12), MLS (12), Fosfomycin (8), Disinfecting agents and antiseptics (6), Aminocoumarin (5), Lincosamide (5), Sulfonamide (4), Streptogramin (3), Mupirocin (3), Antibacterial free fatty acids (2), Bicyclomycin (1), Elfamycin (1), Nitroimidazole (1), Pleuromutilin (1), and Others (6). Our results show that abundant ARGs persistent in sewage, most of which belong to antibiotics commonly used in clinical practice (Figure 2B).

A total of 93 MGE subtypes belonging to 8 MGE types were found in the DWW. Among them, Transposases (25) was the most frequently detected MGE, followed by Plasmids (28), Insertion sequences for Transposases (23), Tn916 transposon (19), Integrases (4), ISCRs (3), Transposition Module (2), and TNP-ISCR (2; in subtypes; Figure 2C).

### Removal efficiency of bacteria, ARGs and MGEs by treatment

**Figure 3:**
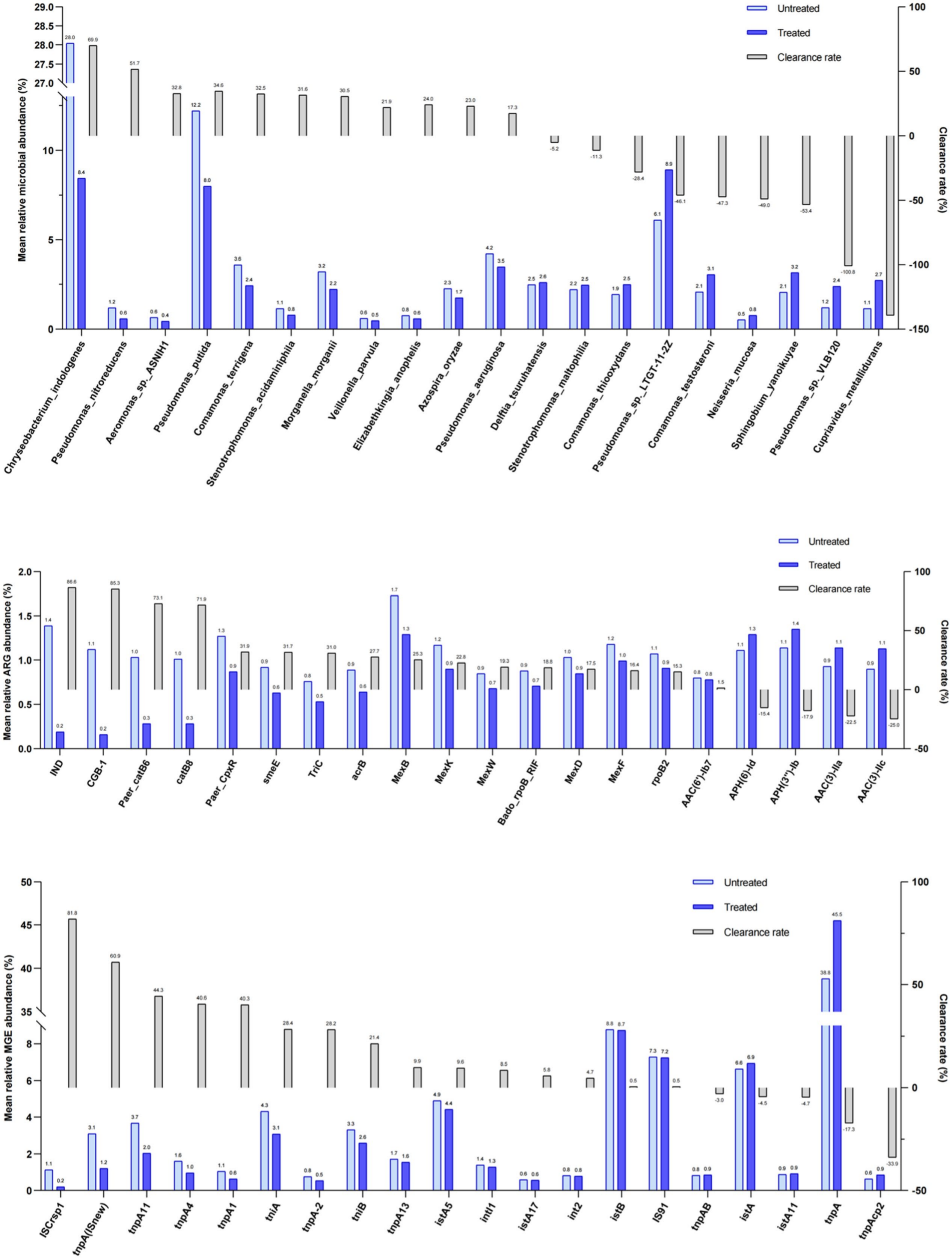
Changes in relative abundance of bacteria, ARGs and MGEs in the untreated and treated dental waste waters and their clearance rate. Light blue and dark blue columns show the mean relative abundance of bacteria **(A)**, ARGs **(B)**, and MGEs**(C)** in the two groups. The gray columns show the corresponding clearance rates.

Our data indicate that, on average, the relative abundance of nine bacteria was reduced by the treatment (see Methods section) of DWW. For example, *Chryseobacterium Indologenes* were reduced from 28.04 to 8.43% and *Pseudomonas putida* from 12.2 to 7.98%. Other reduced bacteria included *Pseudomonas aeruginosa*, *Comamonas terrigena*, *Morganella morganii*, *Azospira oryzae*, *Stenotrophomonas acidaminiphila*, *Pseudomonas nitroreducens*, *Elizabethkingia anopheles*, *Aeromonas* sp. ASNIH1, and *Veillonella parvula*. On the contrary, the average relative abundance of 9 bacteria was slightly increased after treatment, including *Pseudomonas* sp. Ltgt-11-2z, *Cupriavidus metallidurans*, *Pseudomonas* sp. VLB120, *Sphingobium yanoikuyae*, *Delftia tsuruhatensis*, *Afipia broomeae*, *Comamonas testosterone*, *Stenotrophomonas maltophilia*, *Comamonas thiooxydans*, and *Neisseria mucosa*. However, the differences between the treated and untreated waste water samples were not statistically significant (Figure 3A; Supplementary Table 1).

To provide more accurate determination of changes in bacteria between the untreated and treated groups of DWW, LEfSe analysis was used to identify taxa with differential abundance based on bacteria with LDA threshold >2. Our results revealed 29 taxa with significant differences in both groups: 28 were in the untreated samples, mainly *Alphaproteobacteria*, *Gammaproteobacteria*, and *Actinobacteria*, while only *Betaproteobacteria Acidovoraxavenae* in the class *β-Proteobacteria* among the treated samples.

The relative abundance of 20 ARGs were compared in untreated and treated samples: ARGs of Carbapenem and Phenicol were reduced, while ARGs of Cephalosporin were increased by treatment. The ranges of change were larger than that of other types of ARGs although these changes were not significant. Specifically, subtypes of IND, CGB-1, Paer-catB6, and catB8 had higher clearance rate (>70%) through treatment. On the contrary, subtypes of APH(6)-Id, APH (3″)-Ib, AAC(3)-IIa, and AAC(3)-IIc were slightly increased in abundance after treatment (Figure 3B; Supplementary Tables 2–5).

**Figure 4:**
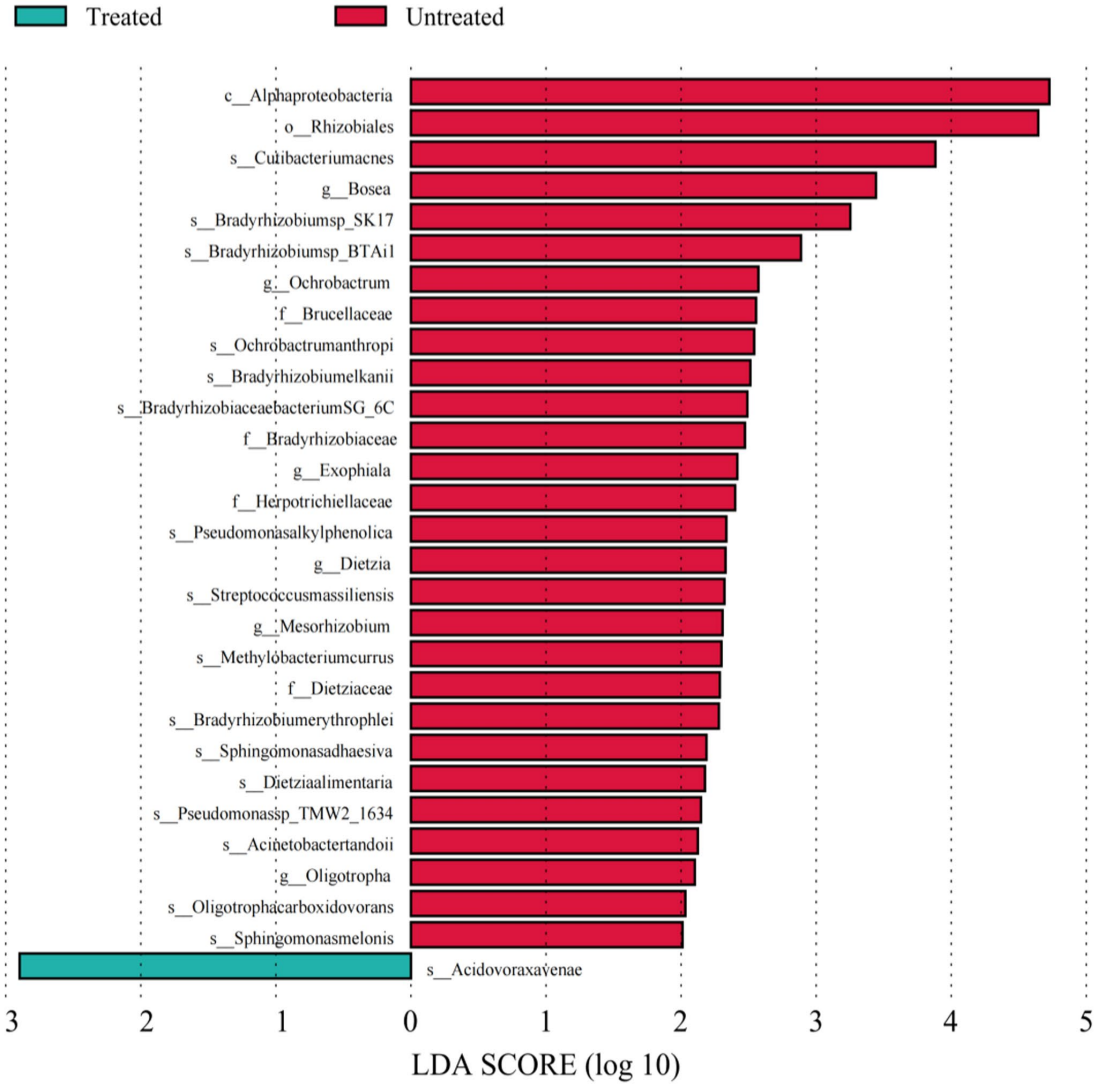
Linear discriminant analyses of bacterial species. Each column represents a bacterium, and the length of the column corresponds to the LDA value. The larger the LDA value, the larger the difference. The color of the bar corresponds to the grouping of characteristic bacteria.

**Figure 5:**
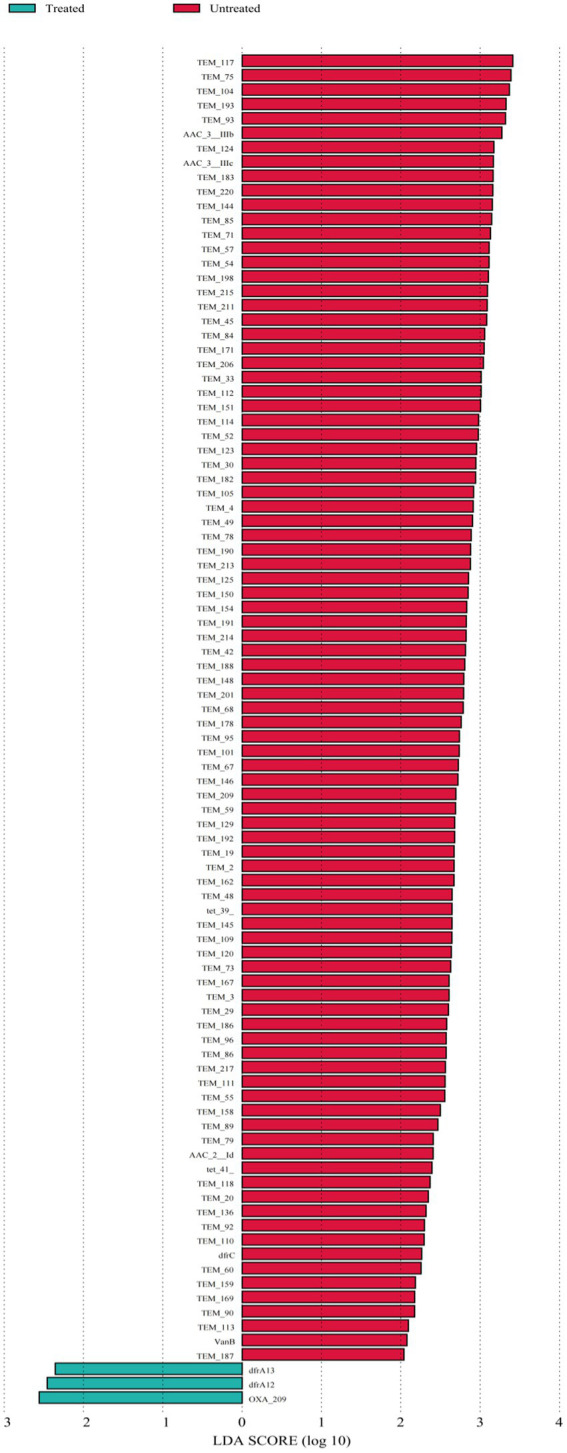
ARGs linear discriminant analyses. Each horizontal column represents an ARG, and the length of the column corresponds to the LDA value. The larger the LDA value, the larger the difference. The color of this bar corresponds to the grouping of feature ARGs.

The differential ARGs and MGEs were evaluated based on LEfSe analysis. The LDA histograms of ARGs and MGEs were presented in Figures 4, 5. The lengths of the bars represent the contribution from different species (LDA Score). The featured ARGs (LDA > 2) were mainly TEM (84 subtypes), tet 39, tet 41, AAC-3 (2 subtypes), AAC-2, VanB, and dfrC in the untreated samples while only dfrA12, dfrA13, and OXA-209 were detected in the treated samples. Among MGEs, only tnpAa (LDA > 2) showed the biggest difference between the treated and untreated samples. The ARG classifications before and after treatment were shown in Figure 5.

### Correlations among bacterial communities and ARGs

**Figure 6:**
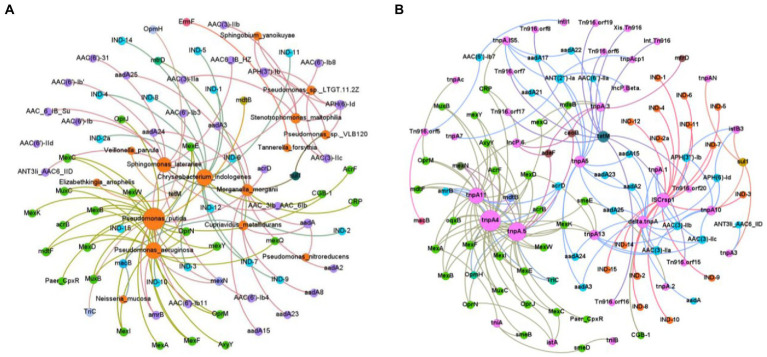
Network analysis of co-occurrence patterns. Each node connection indicates strong (Spearman correlation coefficient ρ > 0.8) and significant (*p* < 0.01) correlation. The size of each node is proportional to the number of connections. **(A)** Co-occurrence network of bacteria and ARG. **(B)** Co-occurrence network of ARGs and MGEs.

To further evaluate correlations between ARGs and the more dominant genera, the top abundant ARGs (100 subtypes) and the top 30 bacterial species were selected for the Spearman correlation coefficients analysis (Langmead and Salzberg, 2012). From the analysis, the positive-strong correlations (*r* > 0.8, *p* < 0.01) were selected for building a network of co-occurrences. A co-occurrence network contained 88 nodes (27 bacteria, 95 antibiotic subtypes) and 101 edges. Among all the bacteria, *Pseudomonas putida*, *Pseudomonas aeruginosa*, *Chryseobacterium indologenes*, *Sphingomonas laterariae* were located in the central hub. In particular, *Pseudomonas putida* correlated with Mex (11 subtypes), OprN/J/M, TriC, OpmH, mdtB/F, AxyY, acrB/D, MuxB/C, AcrF, Paer-CpxR, and amrB; *Pseudomonas aeruginosa* with OprM/N, Mex (10 subtypes), MuxB/C, mdtF, amrB; *Sphingomonas laterariae* with sul1, ANT3Ii-AAC6-IID, AAC-6-IB-Su/-HZ, AAC(6′; 8 subtypes), and AAC-3Ib-AAC-6Ib; *Chryseobacterium indologe* with IND (15 subtypes), and CGB-1; *Sphingomonas laterariae* with sul1, ANT3Ii-AAC6-IID, AAC-6 (11 subtypes), AAC-3Ib and AAC-6Ib; *Cupriavidus metallidurans* with aadA/A8, macB, mtrD; *Morganella morganii* with CRP、aadA (7 subtypes); *Neisseria mucosa* with macB; *Pseudomonas nitroreducens* with aadA; *Pseudomonas* sp. *LTGT.11.2Z* with APH(3″)-Ib, APH(6)-Id, and AAC(3)-IIc; *Pseudomonas sp. VLB120* with sul1, APH(3″)-Ib, and APH(6)-Id; *Sphingobium yanoikuyae* with APH(3″)-Ib and APH(6)-Id; *Stenotrophomonas maltophilia* with AAC(3)-IIb/-IIa/-IIc, APH(3″)-Ib, and APH(6)-Id; *Tannerella forsythia* with ErmF; and *Veillonella parvula* with tetM (Figure 6A).

### Correlations among ARGs and MGEs

Spearman correlation coefficients were used to evaluate correlations between ARGs and MGEs, using the top 100 ARG subtypes and 93 MGE subtypes. The positive-strong correlations (r > 0.8, *p* < 0.01) were selected for a network co-occurrence analysis. The co-occurrence network consisted of 109 nodes and 175 edges. Specifically, ISCrsp1 and tnpA.5/4/11 were located in the central hubs of the network, with the largest number of ARGs connected to them. In addition, ISCrsp1 was mainly correlated with IND (15 subtypes); delta.tnpA with AAC(3; 3 subtypes), aadA (6 subtypes), APH(3″)-Ib, and APH(6)-Id; tnpA.5 with oqxB, Mex (10 subtypes), Paer-CpxR, acrB/D, mdtB/F, AxyY, TriC, adeF, ceoB, OpmH, OprN/J, AcrF, and amrB; tnpA5 with ANT(2″)-Ia, aadA (9 subtypes), and AAC(6′)-IIa; tnpA4 with Mex (12 subtypes), MuxB/C, AcrF, mdtB/F, AxyY, TriC, OpmH, OprM/J/N, acrB/D, amrB, CRP, oqxB, and smeE/B; tnpA11with Mex (9 subtypes), acrB/D/F, MuxB/C, mdtB/F, AxyY, amrB, and OprM; IncP.6. with adeF, ceoB, CRP, oqxB, and mdsB; Tn916 with orf (9 subtypes), and tetM; tnpA10 with APH(3″)-Ib, APH(6)-Id, AAC(3)-IIc/-IIa/-IIb, and sul1(Figure 6B).

## Discussion

Studies on hospital waste waters have shown strong associations between their contaminants (ARG, MGE, and antibiotic resistant microbes) and environmental health problems (Su et al., 2017; Quintela-Baluja et al., 2019; Cai et al., 2021). DWW may contain the similar types of contaminants as found in hospital waste but also a substantial amount of heavy metals which may influence interactions among ARG, MGE and microbes. Since metals increase the potential for ARGs spread *via* co-selection of ARGs and MGEs, co-existence of the metals and ARGs would make the DWW to be a novel niche for studying AMR emergence and environmental health concerns. However, there have been very limited reports on environmental health hazards with DWW, especially with new technology such as resistome and mobilome in our investigation. By using a metagenomics approach, DWW samples from one major dental department were found to have resistome which included 1,208 types of ARGs belonging to 29 antibiotic types/subtypes. The most abundant ones were ARGs of multidrug resistance, followed by ARGs of Aminoglycoside, Cephalosporin, Fluoroquinolone, Tetracycline, Peptide, Cephamycin, Phenicol, Glycopeptide, Carbapenem, Diaminopyrimidine, Rifamycin, Macrolide, Penam, MLS, and Fosfomycin. Importantly, all of the mentioned resistance was to antibiotics which were commonly used in clinical practice in the hospital but were less frequently used in the dental department where the waste water samples were collected. Our results are intriguing as well as meaningful because DWW was thought to be rarely involved in the transmission of AMR. Furthermore, a wide variety of ARGs were unexpectedly found in DWW which might have been influenced by the abundant metals. These unique features need to be further investigation in order to better understand mechanisms and to develop more effective prevention strategies.

Microbiomes have been considered as an important driver for ARG disseminations in the environment (Baym et al., 2016; Jia et al., 2017; Chen et al., 2019; Yu et al., 2020). The source of ARGs in the DWW may come from oral microbiome. Indeed, our collected DWW samples included 1,514 types of bacteria, 37 types of fungi, 11 types of phages, 7 types of Archaea, and 5 types of viruses. Among them, bacteria were the majority while the most abundant phyla and genus were Proteobacteria (76.4%), and Pseudomonas (25.67%), particularly *Pseudomonas putida*, *Pseudomonas* sp. *LTGT-11-2Z*, and *Pseudomonas aeruginosa*. Importantly, they also belonged to the important pathogens found in dental clinic. A previous study revealed that composition of the microbial community in waste water was associated with 68.2% of the variations in ARGs (Zhang et al., 2016). Using the association data from our metagenomic analyses, network and binning analyses were conducted as shown in other reports (Guo et al., 2017; Liu et al., 2019; Sun et al., 2021). Our analyses revealed a complicated co-occurrence network which contained 88 nodes and 101 edges, and which involved 27 bacteria and 95 ARGs subtypes. Specifically, *Pseudomonas putida*, *Pseudomonas aeruginosa*, *Chryseobacterium indologenes*, and *Sphingomonas laterariae* were located in the central hubs of the network and involved with abundant ARGs referring to various antibiotics. Some of the ARBs which were associated with resistance to multiple drugs have been reported to contribute to increased morbidity and mortality among patients (Morrison and Zembower, 2020). Therefore, understanding existence of networks for ARGs and bacteria would provide valuable information in predicting novel ARB and in designing prevention protocols against emerging AMR (Chng et al., 2020).

Mobilome is defined as all detectable HGT elements within a given metagenomic dataset and these elements included plasmids, integrative conjugative elements (ICE), transposons, and insertional repeat sequences (Pal et al., 2015; Ju et al., 2019; Yun et al., 2021). In this study, mobilome of the DWW was composed of 93 MGE subtypes belonging to 8 MGE types. Among them, transposases were the most frequently detected MGEs. With our correlation analyses, most of ARGs showed significant correlations with total abundance of MGEs. The co-occurrence network consisted of 109 nodes and 175 edges. Among all MGEs, ISCrsp1, and tnpA.5/4/11 were located in the central hubs of the network and might serve as links to different ARG types. With the large number of ARGs connected to them, they took on the active role of ARG dissemination. Previous studies indicate that most co-occurring ARGs with metals also co-occurred with MGEs (Song et al., 2017; Zhao et al., 2019).

Heavy metals can promote resistance to antibiotics either *via* cross-resistance (a single genetic unit conferred resistant to both metals and antibiotics), co-resistance (both metal resistant genes (MRGs) and ARGs are associated with same MGEs), or co-regulation (both metal and antibiotic resistance shared their regulatory systems; Baker-Austin et al., 2006; Imran et al., 2019). Moreover, the relative MRGs and ARGs abundances would increase with increasing metals concentration (Hu et al., 2017). The metal-driven selection of AMR is markedly greater when both MRGs and ARGs are situated on the same MGEs (e.g., plasmids, transposons, and integrons; Di Cesare et al., 2016; Hu et al., 2017). For example, int1 has been closely associated with MRG czcA, coding for cobalt (Co), zinc (Zn), and cadmium (Cd) resistance, and beta-lactamase resistance (Stokes et al., 2006; Gillings et al., 2008), indicating that MRGs and ARGs may be transferred simultaneously to host bacteria *via* int1 in the HGT process (Gupta et al., 2022). Transformation is the main pathway of HGT, which can take place in more than 80 naturally competent bacterial species with distant phylogenetical backgrounds, even consisting of human pathogens (Thomas and Nielsen, 2005; Maeusli et al., 2020). Due to the prevalence of extracellular ARGs, antibiotics and naturally competent bacteria, the environmental transformation of ARGs is estimated to be quite frequent and is one of the predominant pathways to spread AMR (de Aldecoa et al., 2017). Ag, CuO and ZnO-based NPs/ions could promote the natural transformation of plasmids harboring ARGs (Zhang et al., 2022), and the promoting effect can occur at clinically relevant concentrations (Hernandez-Sierra et al., 2008; Mohler et al., 2018) or realistic concentrations within aquatic environments (Brunetti et al., 2015). On the other hand, heavy metals pollution has altered bacterial diversity and abundance, as the bacterial population is sensitive to heavy metals (Chen et al., 2018), and the long-term presence of high concentrations of metals in polluted water may increase heavy metal resistance in a variety of bacteria (Gupta et al., 2022). These observations suggest an underlying metal-driven co-selection process which was linked with existence of cross-resistance (Li et al., 2017; Zhao et al., 2019). Furthermore, MGEs are actively involved in HGT of ARGs in neighboring microbial communities (Gupta et al., 2018). Consequently, when DWW are released into the environment, it becomes very difficult to efficiently eliminate the generation of ARB (Rakita et al., 2020).

In our study, the main limitation is that metal concentrations were not determined due to our limitations of analytical techniques. Theoretically, a large amount of heavy metals discharged from clinical practice in every day will inevitably lead to a large amount of heavy metals in DWW. On the other hand, previous reports indicate the presence of high levels of Cu, Zn, Hg and MeHg in DWW (Rani et al., 2015). Thus, co-selection and cross-resistance would occur in DWW. If ARGs and MRG is found on the same MGEs, and this physical linkage results in co-resistance. Cross-resistance is another co-selection mechanism which occurred when single genes encoded resistance to both antibiotics and metals (Li et al., 2017; Zhao et al., 2019). Better understanding of how metals influence formation of ARGs and MGEs would provide insights into novel mechanisms of HGT and emergence of ARB in the future.

The generation and mobility of clinically relevant ARGs in waste waters post significant risk to human health (Carvalho and Santos, 2016; Liu et al., 2018b; Slizovskiy et al., 2020). Our data clearly show the abundance of ARGs and MGEs in the DWW, therefore more effective treatment of the waste water is of great importance. Unfortunately, few existing processes have been designed to remove ARGs, and our data as well as others indicate that such processes were not highly effective (Mao et al., 2015; Bengtsson-Palme et al., 2016; Di Cesare et al., 2016; Pazda et al., 2019; Vinzelj et al., 2020; Li et al., 2022). The treatment process in our dental department utilized ozone, which reacts directly or indirectly *via* a hydroxyl radical mechanism to reduce organic and inorganic materials to become more biodegradable, and which efficiently inactivate a wide range of microorganisms (Tripathi and Tripathi, 2011). Our results show that only a few bacteria with clearance rate higher than 30% were observed. On the contrary, some others were increased. Considering that metagenomics only detects bacterial DNA, the data do not represent activity and integrity of bacteria. Thus, clearance or abundance of bacteria should be reconfirmed *via* bacterial isolates.

As to resistome and mobilome, our data show that ozone treatment had no obvious effects in changing the abundance of resistome and mobilome, although a previous study revealed that antibiotic-resistant hosts and resistant genes were significantly inactivated by ozone treatments (Pei et al., 2016), as well as a significant portion of only MLS and tetracycline genes (Raza et al., 2022). On the other hand, beta lactam ARGs were increased by UV-, chlorine-, and ozone-based treatment strategies (Guo et al., 2013; Alexander et al., 2016; Ferro et al., 2017; Liu et al., 2018a). These different observations are likely due to the use of different experimental designs, sample sizes and technologies. More systematic studies are needed to identify efficacy of waste water treatment protocols.

ARGs and MGEs have been listed as serious and emerging environmental pollutants and health problems from hospital waste waters (Gillings et al., 2008; Xu et al., 2016; Chen et al., 2019). Our data strengthened the addition of DWW to the list of concerns. Our study reveals that DWW harbored a significant and diversity of microbes, ARGs, and MGEs, providing a persistent selection pressure (in the presence of heavy metals) and possibly resulting in the occurrence or emergence of novel antimicrobial determinants. Our observations provide evidence which underscore the need for improved disinfection methods, and for monitoring the waste prior to disposal. Effective effort would lead to reducing the spread of drug resistant bacteria into hospitals and communities.

## Author’s note

The proposal was approved by the institutional review board of the First Affiliated Hospital of Shantou University Medical College.

## Data availability statement

The dataset presented in this study are deposited and can be found in an online repository. The name of the repository and accession number(s) can be found below: NCBI repository - https://www.ncbi.nlm.nih.gov/, PRJNA869027.

## Ethics statement

The studies involving human participants were reviewed and approved by First Affiliated Hospital of Shantou University Medical College. Written informed consent for participation was not required for this study in accordance with the national legislation and the institutional requirements.

## Author contributions

XJ and WG contributed equally to this manuscript. YX and WG carried out the sample collection, experimental studies, and drafted the manuscript. FYa, QX, LC, FYu, and PY participated in the innovative design of the study. XL, MZ, YY, XG, and MW, performed the statistical analysis. QZ and XJ conceived the study, participated in its design and coordination, and helped to revise the manuscript. All authors contributed to the article and approved the submitted version.

## Conflict of interest

The authors declare that the research was conducted in the absence of any commercial or financial relationships that could be construed as a potential conflict of interest.

## Publisher’s note

All claims expressed in this article are solely those of the authors and do not necessarily represent those of their affiliated organizations, or those of the publisher, the editors and the reviewers. Any product that may be evaluated in this article, or claim that may be made by its manufacturer, is not guaranteed or endorsed by the publisher.
