# Supplementary material for: New insight into the microbiome, resistome, and mobilome on the dental waste water in the context of heavy metal environment
Source: Front Microbiol. 2023 Apr 20;14:1106157. doi: 10.3389/fmicb.2023.1106157 (PMC10157219; doi:10.3389/fmicb.2023.1106157)
Supplement: Supplementary file 1 [file Data_Sheet_1.docx]

Supplement Table 1. Differences in relative abundance of the TOP 20 bacterial species in untreated and treated dental waste waters.

| **Supplement Table 1 Differences in relative abundance of TOP 20 bacterial species in untreated and treated dental waste waters** | | | |
| --- | --- | --- | --- |
| Species type | Untreated (%) (Mean±SD) | Treated (%) (Mean±SD) | p value |
| *Chryseobacterium indologenes* | 28.04±29.208 | 8.43±8.564 | 0.413 |
| *Pseudomonas putida* | 12.20±9.143 | 7.98±3.563 | 0.556 |
| *Pseudomonas* sp. *LTGT-11-2Z* | 6.10±3.067 | 8.91±5.600 | 0.413 |
| *Pseudomonas aeruginosa* | 4.21±5.396 | 3.48±2.448 | 0.73 |
| *Comamonas terrigena* | 3.60±2.132 | 2.43±1.298 | 0.556 |
| *Morganella morganii* | 3.21±3.436 | 2.23±2.600 | 0.905 |
| *Sphingobium yanoikuyae* | 2.06±1.037 | 3.16±1.813 | 0.286 |
| *Delftia tsuruhatensis* | 2.48±1.800 | 2.61±1.389 | 1 |
| *Afipia broomeae* | 0.09±0.083 | 5.56±8.972 | 0.063 |
| *Comamonas testosterone* | 2.07±0.713 | 3.05±1.198 | 0.19 |
| *Stenotrophomonas maltophilia* | 2.21±1.628 | 2.46±1.512 | 0.413 |
| *Comamonas thiooxydans* | 1.94±0.986 | 2.49±1.125 | 0.556 |
| *Azospira oryzae* | 2.26±3.845 | 1.74±2.841 | 0.556 |
| *Cupriavidus metallidurans* | 1.14±0.905 | 2.73±2.929 | 0.556 |
| *Pseudomonas* sp. *VLB120* | 1.19±0.450 | 2.39±1.956 | 0.413 |
| *Stenotrophomonas acidaminiphila* | 1.14±0.647 | 0.78±0.287 | 0.413 |
| *Pseudomonas nitroreducens* | 1.18±1.920 | 0.57±0.422 | 0.905 |
| *Elizabethkingia anopheles* | 0.75±0.985 | 0.57±0.423 | 0.73 |
| *Neisseria mucosa* | 0.51±0.439 | 0.76±0.801 | 0.905 |
| Others | 23.64±17.119 | 37.68±10.480 | 0.286 |

Supplement **Table 2.** Differences in relative abundance of the TOP 20 ARGs in untreated and treated dental waste waters

| **Supplement Table 2 Differences in relative abundance of TOP 20 ARGs in untreated and treated dental waste waters** | | | |
| --- | --- | --- | --- |
| ARG type | Untreated (%) (Mean±SD) | Treated (%) (Mean±SD) | p value |
| Aminoglycoside | 18.64±3.796 | 22.42±11.783 | 0.413 |
| Carbapenem | 15.54±17.506 | 2.27±2.028 | 0.556 |
| Phenicol | 6.47±2.497 | 2.80±1.542 | 0.063 |
| Cephalosporin | 2.12±1.805 | 8.17±10.276 | 0.413 |
| Tetracycline | 2.95±2.848 | 2.61±1.262 | 0.73 |
| Peptide | 3.24±2.417 | 1.58±1.249 | 0.413 |
| Fluoroquinolone | 2.38±2.538 | 1.42±0.656 | 0.73 |
| Rifamycin | 2.06±0.866 | 1.81±0.698 | 0.905 |
| Cephamycin | 1.82±1.759 | 1.20±0.856 | 1 |
| Disinfecting agents and antiseptics | 1.76±1.062 | 1.18±0.937 | 0.413 |
| MLS | 0.93±0.853 | 0.66±0.329 | 0.905 |
| Aminocoumarin | 0.85±0.360 | 0.72±0.444 | 0.556 |
| Sulfonamide | 0.66±0.335 | 0.93±0.394 | 0.286 |
| Macrolide | 0.69±0.494 | 0.50±0.402 | 0.413 |
| Diaminopyrimidine | 0.40±0.257 | 0.12±0.072 | 0.063 |
| Glycopeptide | 0.09±0.058 | 0.42±0.626 | 0.556 |
| Fosfomycin | 0.25±0.171 | 0.19±0.138 | 0.73 |
| Nitroimidazole | 0.22±0.140 | 0.17±0.096 | 0.73 |
| Antibacterial free fatty acids | 0.15±0.127 | 0.12±0.145 | 0.413 |
| Mupirocin | 0.06±0.057 | 0.07±0.062 | 0.413 |
| Lincosamide | 0.06±0.084 | 0.06±0.082 | 0.73 |
| Streptogramin | 0.10±0.103 | 0.01±0.017 | 0.286 |
| Bicyclomycin | 0.06±0.102 | 0.04±0.042 | 0.905 |
| Penam | 0.03±0.072 | 0.03±0.037 | 0.73 |
| Pleuromutilin | 0.01±0.010 | 0.01±0.008 | 0.73 |
| Elfamycin | 0.0002±0.0004 | 0.00±0.00 | 0.73 |
| Dual | 6.91±3.847 | 5.63±3.210 | 0.556 |
| Multiple | 30.68±11.447 | 44.03±18.549 | 0.413 |

Supplement **Table 3.** Differences in relative abundance of MGEs in untreated and treated dental waste waters

| **Supplement Table 3 Differences in relative abundance of MGEs in untreated and treated dental waste waters** | | | |
| --- | --- | --- | --- |
| MGE type | Untreated (%) (Mean±SD) | Treated (%) (Mean±SD) | p value |
| Transposases | 54.65±7.725 | 56.56±10.152 | 0.73 |
| Insertion sequences for Transposases | 31.21±6.733 | 31.00±13.771 | 0.73 |
| Transposition module | 7.23±2.061 | 5.55±1.991 | 0.413 |
| Tn916 transposon | 2.43±3.371 | 3.71±3.540 | 0.413 |
| Integrases | 2.45±0.833 | 2.32±0.864 | 0.905 |
| ISCRs | 1.15±1.202 | 0.22±0.201 | 0.413 |
| Plasmids | 0.85±0.500 | 0.59±0.459 | 0.413 |
| Tnp-ISCR | 0.03±0.017 | 0.04±0.039 | 0.73 |

Supplement **Table 4.** Bacterial clearance rate of bacteria after waste water treatments

| **Supplement Table 4. Clearance rate of bacteria after waste water treatments** | | | | | | |  |
| --- | --- | --- | --- | --- | --- | --- | --- |
| Species | Mean relative microbial abundance (%)  Untreated Treated  (n=5) (n=4) | | | Clearance rate p value | |  | |
| *Chryseobacterium indologenes* | 28.04 | 8.43 | 69.94% | | 0.413 | |  |
| *Pseudomonas nitroreducens* | 1.18 | 0.57 | 51.69% | | 0.905 | |  |
| *Aeromonas* sp.*ASNIH1* | 0.64 | 0.43 | 32.81% | | 0.462 | |  |
| *Pseudomonas putida* | 12.20 | 7.98 | 34.59% | | 0.556 | |  |
| *Comamonas terrigena* | 3.60 | 2.43 | 32.50% | | 0.556 | |  |
| *Stenotrophomonas acidaminiphila* | 1.14 | 0.78 | 31.58% | | 0.413 | |  |
| *Morganella morganii*  *Veillonella parvula* | 3.21  0.59 | 2.23  0.46 | 30.53%  21.91% | | 0.905  0.556 | |  |
| *Elizabethkingia anophelis* | 0.75 | 0.57 | 24.00% | | 0.73 | |  |
| *Azospira oryzae* | 2.26 | 1.74 | 23.01% | | 0.556 | |  |
| *Pseudomonas aeruginosa* | 4.21 | 3.48 | 17.34% | | 0.73 | |  |
| *Delftia tsuruhatensis* | 2.48 | 2.61 | -5.24% | | 1 | |  |
| *Stenotrophomonas maltophilia* | 2.21 | 2.46 | -11.31% | | 0.413 | |  |
| *Comamonas thiooxydans* | 1.94 | 2.49 | -28.35% | | 0.556 | |  |
| *Pseudomonas* sp. *LTGT-11-2Z* | 6.10 | 8.91 | -46.07% | | 0.413 | |  |
| *Comamonas testosteroni* | 2.07 | 3.05 | -47.34% | | 0.19 | |  |
| *Neisseria mucosa* | 0.51 | 0.76 | -49.02% | | 0.905 | |  |
| *Sphingobium yanoikuyae* | 2.06 | 3.16 | -53.40% | | 0.286 | |  |
| *Pseudomonas* sp. *VLB120* | 1.19 | 2.39 | -100.84% | | 0.413 | |  |
| *Cupriavidus metallidurans* | 1.14 | 2.73 | -139.47% | | 0.556 | |  |

Supplement **Table 5.** The clearance rate of ARGs after waste water treatments

| **Supplement Table 5 Clearance rate of ARGs after waste water treatments** | | | | |
| --- | --- | --- | --- | --- |
| ARG subtype | Mean relative ARG abundance (%)  Untreated Treated  (n=5) (n=4) | | Clearance rate | *P* Value |
| IND | 1.39 | 0.19 | 86.61% | 0.462 |
| CGB-1 | 1.12 | 0.16 | 85.32% | 0.286 |
| Paer_catB6 | 1.03 | 0.28 | 73.05% | 0.111 |
| catB8 | 1.01 | 0.28 | 71.88% | 0.111 |
| Paer_CpxR | 1.27 | 0.87 | 31.93% | 0.462 |
| smeE | 0.92 | 0.63 | 31.72% | 0.221 |
| TriC | 0.76 | 0.53 | 30.98% | 0.413 |
| acrB | 0.89 | 0.64 | 27.69% | 0.327 |
| MexB | 1.73 | 1.29 | 25.26% | 0.462 |
| MexK | 1.17 | 0.9 | 22.84% | 0.462 |
| MexW | 0.85 | 0.68 | 19.32% | 0.462 |
| Bado_rpoB_RIF | 0.88 | 0.71 | 18.81% | 0.806 |
| MexD | 1.03 | 0.85 | 17.50% | 0.624 |
| MexF | 1.18 | 0.99 | 16.35% | 0.624 |
| rpoB2 | 1.07 | 0.91 | 15.27% | 0.806 |
| AAC(6')-Ib7 | 0.8 | 0.78 | 1.52% | 0.806 |
| APH(6)-Id | 1.11 | 1.29 | -15.44% | 0.462 |
| APH(3'')-Ib | 1.14 | 1.35 | -17.94% | 0.462 |
| AAC(3)-IIa | 0.93 | 1.14 | -22.50% | 0.462 |
| AAC(3)-IIc | 0.9 | 1.13 | -24.97% | 0.327 |

Supplement **Table 6.** The clearance rate of MGEs after waste water treatments

| **Supplement Table 6 Clearance rate of MGEs after waste water treatments** | | | | | | |  |
| --- | --- | --- | --- | --- | --- | --- | --- |
| MGE subtype | Mean relative MGE abundance (%)  Untreated Treated  (n=5) (n=4) | | Clearance rate | | *P* Value |  |  |
| ISCrsp1 | 1.13 | 0.20 | | 81.84% | 0.221 | | |
| tnpA(ISnew) | 3.11 | 1.21 | | 60.93% | 0.327 | | |
| tnpA11 | 3.67 | 2.04 | | 44.33% | 0.327 | | |
| tnpA4 | 1.61 | 0.96 | | 40.64% | 0.142 | | |
| tnpA1 | 1.05 | 0.63 | | 40.30% | 0.086 | | |
| tniA | 4.32 | 3.09 | | 28.36% | 0.142 | | |
| tnpA-2 | 0.76 | 0.54 | | 28.21% | 0.462 | | |
| tniB | 3.31 | 2.60 | | 21.35% | 0.462 | | |
| tnpA13 | 1.72 | 1.55 | | 9.89% | 1 | | |
| istA5 | 4.90 | 4.43 | | 9.59% | 0.462 | | |
| intI1 | 1.39 | 1.28 | | 8.51% | 1 | | |
| istA17 | 0.59 | 0.56 | | 5.82% | 0.624 | | |
| int2 | 0.82 | 0.78 | | 4.70% | 0.806 | | |
| istB | 8.78 | 8.74 | | 0.53% | 1 | | |
| IS91 | 7.28 | 7.24 | | 0.48% | 1 | | |
| tnpAB | 0.83 | 0.85 | | -3.00% | 1 | | |
| istA | 6.64 | 6.94 | | -4.48% | 0.806 | | |
| istA11 | 0.88 | 0.92 | | -4.70% | 0.462 | | |
| tnpA | 38.79 | 45.50 | | -17.32% | 0.221 | | |
| tnpAcp2 | 0.63 | 0.85 | | -33.86% | 0.327 | | |
